# Frequency-specific static and dynamic neural activity indices in children with different attention deficit hyperactivity disorder subtypes: a resting-state fMRI study

**DOI:** 10.3389/fnhum.2024.1412572

**Published:** 2024-08-09

**Authors:** Ran Chen, Yun Jiao, Jun-Sa Zhu, Xun-Heng Wang, Mei-Ting Zhao

**Affiliations:** ^1^Nurturing Center of Jiangsu Province for State Laboratory of AI Imaging and Interventional Radiology, Department of Radiology, Zhongda Hospital, Medical School of Southeast University, Nanjing, China; ^2^Network Information Center, Zhongda Hospital, Medical School of Southeast University, Nanjing, China; ^3^Institute of Biomedical Engineering and Instrumentation, Hangzhou Dianzi University, Hangzhou, China; ^4^Department of Radiology, Nanjing BenQ Medical Center, the Affiliated BenQ Hospital of Nanjing Medical University, Nanjing, China

**Keywords:** attention deficit hyperactivity disorder, resting-state fMRI, ADHD subtype, frequency band, temporal dynamics

## Abstract

Attention deficit hyperactivity disorder (ADHD) is one of the most common neurodevelopmental disorders in childhood. Numerous resting-state functional magnetic resonance imaging (rs-fMRI) studies in ADHD have been performed using traditional low-frequency bands (0.01–0.08 Hz). However, the neural activity patterns of frequency subbands in ADHD still require further investigation. The purpose of this study is to explore the frequency-dependent characteristics and neural activity patterns of ADHD subtypes. We selected the ADHD combined type (ADHD-C, *N* = 25), ADHD inattentive type (ADHD-I, *N* = 26) and typically developing (TD, *N* = 28) children from the ADHD-200 Consortium. Based on the slow-5 band (0.01–0.027 Hz) and slow-4 band (0.027–0.073 Hz), we generated static and dynamic fractional amplitude of low-frequency fluctuation (fALFF) and regional homogeneity (ReHo) maps for each participant. A flexible-factorial analysis of variance model was performed on static and temporal dynamic rs-fMRI measurements within two subbands. Results revealed that the orbital-frontal gyrus, precuneus, superior temporal gyrus and angular gyrus were found to have obvious frequency band and group interaction effects. The intrinsic neural activity differences among three groups were more prominent in the slow-5 frequency band compared to the slow-4 band. In addition, the indices of significant interaction regions showed correlations with the progression of the disease and the features in slow-5 showed an advantageous diagnostic performance compared with those in slow-4. The results suggested the intrinsic neural activities of ADHD subtypes were frequency-dependent. The frequency-specific analysis of static and dynamic brain activity may provide a deeper understanding of neurophysiological dysfunction patterns in ADHD subtypes and provide supplementary information for assessing ADHD subtypes.

## 1 Introduction

Attention deficit hyperactivity disorder (ADHD) is one of the most common neurodevelopmental disorders in childhood. Patients are characterized as having inattention and/or hyperactivity-impulsivity symptoms, which often exert a negative effect on the quality of their social and academic activities. In China, the total prevalence of ADHD in children and adolescents is 6.26%. In addition, the prevalence rate of ADHD in males is significantly higher than that in females (Wang T. et al., 2017). ADHD has a high degree of heritability and significant familial aggregation (Faraone and Larsson, 2019; Tistarelli et al., 2020), which may be related to genetic or environmental factors (Rhee et al., 1999). Although the symptoms of ADHD partially decrease with age, more than half of cases persist into adulthood (Faraone et al., 2006).

The fifth editions of the Diagnostic and Statistical Manual of Mental Disorders (DSM-V) published by the American Psychiatric Association (APA) classified ADHD into different subtypes: inattentive presentation (ADHD-I), hyperactive-impulsive presentation (ADHD-HI) and combined presentation (ADHD-C). The prevalence of ADHD-I is the highest among the subtypes, followed by ADHD-C and ADHD-HI (Wang T. et al., 2017). In the latent class analysis (LCA), the quantitative analysis of ADHD subtypes in different periods emphasized that the symptoms of ADHD would change with the physical development of patients (Krasner et al., 2018). Due to the strong heterogeneity among ADHD individuals (Lahey et al., 2005), it is necessary to monitor and investigate characteristic neurophysiological activities of ADHD subtypes.

Functional magnetic resonance imaging (fMRI), including resting-state and task-state imaging, is a well-known technique for measuring brain function and has been widely used to explore the pathophysiological mechanism of ADHD. Structural imaging (Ellison-Wright et al., 2008; Frodl and Skokauskas, 2012) and task-state imaging (task-fMRI) (Cortese et al., 2012; McCarthy et al., 2014; Lei et al., 2015) were used in early studies of ADHD. Task-fMRI included a variety of tasks in the form of go/no-go, stop-signal or n-back, but multiple meta-analyses showed that the results did not show good consistency (Samea et al., 2019). Resting-state fMRI (rs-fMRI) has gradually garnered attention and it provides a more convenient method compared to task- fMRI (Cortese et al., 2021). For example, psychiatric patients, especially preschool children, are often unable to cooperate with the assigned task, while rs-fMRI can be performed when the patient is lying still without performing tasks. Rs-fMRI is designed to examine spontaneous neural fluctuations in the resting state and is a noninvasive tool for obtaining information on brain function.

To characterize the fluctuating patterns of brain activity, various voxel-based rs-fMRI indices have been proposed. ALFF/fALFF (Zang et al., 2007; Zou et al., 2008) and ReHo (Zang et al., 2004) are among the most commonly used functional indices in fMRI studies. ALFF is defined as the average power spectrum of the time series after Fourier transform in a particular low-frequency band (Zang et al., 2007), and fALFF is the ratio of the power spectrum in the low-frequency band to the entire frequency range (Zou et al., 2008). ALFF reflects the intensity of neural activity by the amplitude of the spectrum, while fALFF represents the relative contribution of a particular oscillation to the entire detectable frequency range. ReHo is a method for calculating time series between a particular voxel and its nearest neighboring voxels (Zang et al., 2004). ReHo reflects the degree of concordance in regional neural activity. They have been widely applied to ADHD patients to reveal aberrant neural activities. Previous studies found that, compared to healthy controls, ADHD patients showed decreased ALFF in the left frontal gyrus, while increased ALFF in the right dorsal superior frontal gyrus (Li et al., 2014). ReHo are observed to show widely-distributed differences in the fronto-cingulo-occipito-cerebellar circuitry (An et al., 2013). Additionally, ALFF/fALFF and ReHo measures are data-driven and might not be biased by factors such as seed definition in seed-based correlation analysis and component selection in independent component analysis. In addition, fALFF is reported to show less susceptible to nuisance noise and higher sensitivity and specificity than ALFF (Zou et al., 2008; Yan et al., 2013). The static measure of fMRI indices assume that the functional activity of brain regions remains stationary throughout the entire resting-state scan. In addition to static rs-fMRI measurements, evidence has shown that spontaneous neuronal activity shows dynamic fluctuations in the resting state (Calhoun et al., 2014; Preti et al., 2017). The dynamic measures estimate the temporal fluctuation patterns of interregional neural interactions (Hutchison et al., 2013; Lurie et al., 2020). A report utilized temporal dynamic rs-fMRI indices in ADHD patients and found a decreased variability in dynamic ALFF in the middle frontal gyrus (Lou et al., 2021). Dynamic functional network analysis revealed that ADHD patients exhibit distinct interactive states in static and dynamic functional connectivity (Ahmadi et al., 2021).

The spontaneous low-frequency oscillations (LFO) of the blood oxygen level-dependent (BOLD) signal measured in rs-fMRI typically are characterized in the range of 0.01–0.08 Hz, which are considered may reflect spontaneous neuronal activity (Penttonen and Buzsáki, 2003). Physiological noise such as the respiration rate and heart rate is recorded within the range of 0.1–0.3 Hz and 0.6–1.1 Hz, respectively (Cordes et al., 2001). In addition, Buzsáki et al. divided the neural oscillation frequency in the brain into multiple subfrequency bands and found that the oscillation within disparate frequency bands may related to different neural processes (Buzsáki and Draguhn, 2004). A study on functional connectivity found that the strengths of functional connections attenuate at different rates as frequency increases across multiple networks (Wang Y. et al., 2020). The LFO can be subdivided into slow-5 (0.01–0.027 Hz) and slow-4 (0.027–0.073 Hz) (Buzsáki and Draguhn, 2004). Studies reported that the amplitude of LFO in gray matter is higher than in white matter and the contributions of slow-4 and slow-5 to LFO amplitudes were different in brain regions such as the precuneus, basal ganglia and thalamus in healthy subjects (Zuo et al., 2010). Xue et al. replicated the experiment and obtained similar findings (Xue et al., 2014). Furthermore, previous studies have shown that Alzheimer’s disease (Yang et al., 2020) and Parkinson’s disease (Wang Z. et al., 2020) exhibit discrepant neural activity in slow-5 and slow-4 bands, suggesting frequency-dependent alterations in the amplitude patterns of mental disorders. However, to the best of our knowledge, few studies have focused on the neural activity patterns of ADHD-C and ADHD-I subtypes in specific subfrequency bands. It is currently urgent to clarify the discrepancy of frequency characteristics in ADHD subtypes. Finer frequency-dependent features may provide important implications and help quantify neural functions in the brain. The approach may hold the potential to help explore the mechanisms driving the differences between subtypes of ADHD.

We aimed to identify the representative neural activity patterns and frequency-dependent characteristics of ADHD-C and ADHD-I subtypes. We utilized both static and dynamic measurements of fALFF and ReHo indices to investigate frequency-related characteristics within the slow-5 and slow-4 bands. We expected to improve the understanding of neurophysiological dysfunction patterns in ADHD subtypes.

## 2 Materials and methods

### 2.1 Participants

**FIGURE 1:**
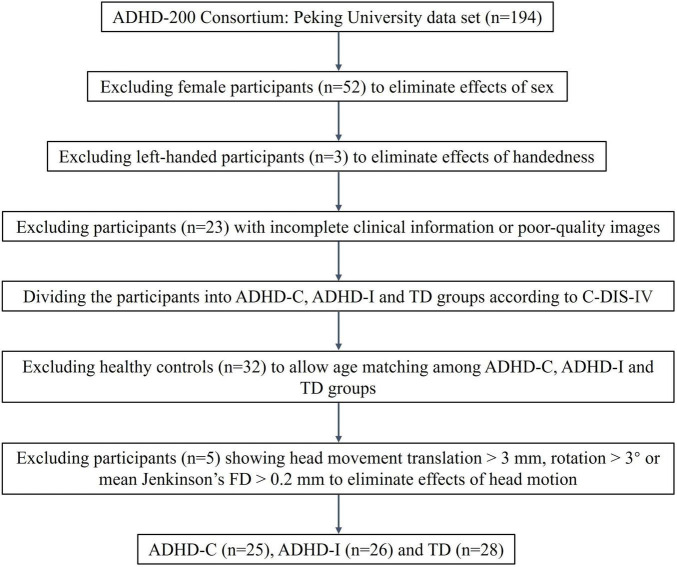
The flowchart of data exclusion.

In the present study, data were collected from the ADHD-200 Consortium (HD-200 Consortium, 2012). The dataset contains data from eight sites and the consortium is committed to facilitating research on the neural basis of ADHD. Access to these data was approved by the research ethics review committees of the respective institutions, and all participants or legal guardians signed informed consent forms before participating. A previous study showed that the cohorts in ADHD-200 were highly inconsistent, and the author suggested that future studies should be conducted using pooled or single cohort data (Wang J. et al., 2017). Therefore, we selected data from one site at Peking University, which possessed more subtypes and more complete information. All participants were assessed using the Computerized Diagnostic Interview Schedule IV (C-DIS-IV), a structured clinical interview of computerized version to provide diagnoses of major psychiatric disorders based on the DSM-IV criteria. They were also evaluated using the Schedule of Affective Disorders and Schizophrenia for Children—Present and Lifetime Version (K-SADS-PL). It is a semistructured interview administered by trained assessors, that contains the screening interview and diagnostic supplement to evaluate comorbid psychiatric disorders. The participants underwent MRI scanning using Siemens 3T Trio scanners, and the scan parameters used were 2000 ms for the repetition time (TR) and 30 ms for the echo time (TE). The acquired voxel size was 3 mm and 4.5 mm. The dataset contains three groups, and the scanning parameters of two groups were identical, but one group had slightly different parameters. More detailed scanning parameters can be found in http://fcon_1000.projects.nitrc.org/indi/adhd200/. We excluded female and left-handed individuals to eliminate the effects of sex and handedness factors on the study. We excluded participants with poor-quality images and incomplete clinical information. Then, based on the DSM-IV diagnostic criteria at enrollment, we divided participants into three categories: ADHD-I, ADHD-C and typically developing (TD) groups. We matched the three groups by age. Participants with excessive head movement (translation > 3 mm, rotation > 3°) and mean Jenkinson’s framewise displacement (FD) (Jenkinson et al., 2002) greater than 0.2 mm were excluded. Finally, we included 25 ADHD-C, 26 ADHD-I and 28 healthy participants. The flowchart of data exclusion was shown in Figure 1.

### 2.2 Data preprocessing

The Data Processing Assistant for Resting-State fMRI (DPARSF, RRID:SCR_002372) (Chao-Gan and Yu-Feng, 2010) was used to preprocess resting-state fMRI data based on Statistical Parametric Mapping (SPM, RRID:SCR_007037) and the Resting-State fMRI Data Analysis Toolkit V1.8 (REST 1.8, RRID:SCR_009641) (Song et al., 2011). (1) The first 10 time points of the imaging data were excluded to avoid signal instability as the participants adapted to the fMRI environment. (2) Time sequence correction was performed to rearrange the images in time and space. (3) Head movement correction was conducted (participants with head translational movement > 3 mm or rotation > 3°, and mean Jenkinson’s FD > 0.2 mm were excluded). (4) Individual structural images were adjusted to the mean functional images for registration. (5) Nuisance covariates, including linear drift, Friston-24 motion parameters, white matter signals and cerebrospinal fluid signals, were regressed out. (6) Functional images were normalized from the original space to the standard space (Montreal Neurological Institute, MNI) with 3 mm isotropic voxels.

The fALFF and ReHo were calculated based on the whole-brain voxel analysis. The calculation method for fALFF was based on the approach proposed by Zou et al. (2008), where it is obtained by the ratio of the power spectrum in the specific frequency band (slow-5 and slow-4 obtained through band-pass filtering in the study) to the entire frequency range (without band-pass filtering). ReHo was measured in both slow-5 and slow-4 bands by calculating Kendall’s coefficient of concordance (KCC), which calculated the similarity of time series between a given voxel and its nearest neighboring voxel (Zang et al., 2004). The criterion for the trueness of adjacent voxels was cornered connections with 26 adjacent voxels. Therefore, the static fALFF and ReHo maps were obtained within the slow-5 and slow-4 bands, respectively. All the fMRI maps were then Z-standardized and smoothed with a 4 mm full width at half maximum Gaussian kernel (FWHM).

Subsequently, as an approach to evaluate the characteristics of the temporal variation in the aforementioned measurements, we used the sliding window method to calculate dynamic fALFF and ReHo indices with Hamming windows. The window length was 48 TR, and the step size was 4 TR. Previous studies showed that different window lengths do not produce significantly different results (Deng et al., 2016; Preti et al., 2017). Moreover, the step size does not significantly affect the variability of fMRI dynamic characteristics, but the empirical value is equal to one-tenth of the window length (Liao et al., 2019). More importantly, we varied the window length (32 TR/64 TR) to validate the reliability of the results. The results of dynamic indices have been included in the Supplementary materials. The voxel-wised fALFF/ReHo indices were calculated in each window and each voxel was assigned one fMRI value in each window. To measure the variability of the dynamic indices over time, the mean and standard deviation (SD) maps across time windows were computed for each index. Finally, the mean and SD maps were Z-standardized and a 4 mm FWHM was used for smoothing.

### 2.3 Statistical analysis

Age, IQ and ADHD-related clinical characteristics were assessed by performing normality and homogeneity of variance tests. Then, demographic and clinical characteristics of ADHD-C, ADHD-I and TD groups were compared by analysis of variance (ANOVA). These statistical analyses were conducted using the IBM SPSS Statistics 26.0 software package (RRID: SCR_016479).

We designed a flexible-factorial ANOVA model to perform voxel-based two-way analysis based on fALFF and ReHo maps using the SPM software package. The model included three groups (ADHD-C, ADHD-I and TD) as between-subject factors and frequency bands as (slow-5 and slow-4) as within-subject factors to observe the effects of interactions between subtypes and frequencies. Age and mean FD were added as covariates and the mask was constructed jointly from the gray matter regions of 90% of the participants. All the statistical maps were corrected for multiple comparisons by using Gaussian Random Field (GRF) correction, combining voxel-wised thresholding *p* < 0.001 and cluster-wised thresholding *p* < 0.05. Then, we extracted regions showing subtype × frequency interaction effects as regions of interest (ROIs). A post-hoc analysis was performed on ROIs to investigate simple effects of fMRI measures among groups and frequency bands. The post-hoc results were corrected for multiple comparisons by Bonferroni correction with *p* < 0.05.

We verified the relationship between ROI signals and disease severity of ADHD-C, ADHD-I and TD. Partial correlation analysis was conducted between fMRI indices and clinical scores of three groups with age and FD as the nuisance covariates. Furthermore, we examined the efficiency of ROIs in classifying ADHD-C, ADHD-I and TD groups, respectively. We compared the features of ROIs in the slow-5 and slow-4 bands. The classification efficiency was verified by plotting the receiver operating characteristic (ROC) curve using a logistic regression model. The area under the ROC curve (AUC) and accuracy were calculated.

## 3 Results

### 3.1 Demographic and clinical characteristics

Demographic and clinical information was represented in Table 1. The results showed no between-group variance in age and verbal IQ. The between-group comparison of diagnostic index was significant, except for ADHD-C and ADHD-I, which showed no difference in the Inattentive Index. Both ADHD subtypes showed a significant reduction in performance IQ compared with the TD group, but no differences were observed between ADHD-C and ADHD-I groups.

**TABLE 1:** Demographic and clinical information of participants.

	ADHD-C	ADHD-I	TD	ANOVA^a^	Between-group tests^b^		
					C-I	C-TD	I-TD
*N*	25	26	28	–		–	
Gender(male)	25	26	28	–		–	
Age(years)	11.6 ± 1.7	12.5 ± 1.7	12.1 ± 1.5	0.148	0.154	0.952	0.935
ADHD Index	56.7 ± 7.5	46.0 ± 6.4	28.5 ± 5.9	<0.001	<0.001	<0.001	<0.001
Inattentive Index	29.6 ± 3.5	27.8 ± 3.2	15.7 ± 3.5	<0.001	0.239	<0.001	<0.001
Hyper/Impulsive Index	27.2 ± 5.2	18.3 ± 4.4	12.8 ± 3.5	<0.001	<0.001	<0.001	<0.001
Verbal IQ	115.4 ± 17.5	110.5 ± 14.7	118.0 ± 13.7	0.198	0.771	0.989	0.228
Performance IQ	100.3 ± 12.4	95.2 ± 16.6	110.6 ± 15.5	0.001	0.697	0.044	0.001

Data are presented as the mean ± standard deviation. C, ADHD-Combined; I, ADHD-Inattentive; TD, typically developing.

^a^*p*-values for ANOVA among the three groups;

^b^*p*-values for between-group effects after Bonferroni multiple comparison correction.

### 3.2 Subtype × frequency interaction effect

**FIGURE 2:**
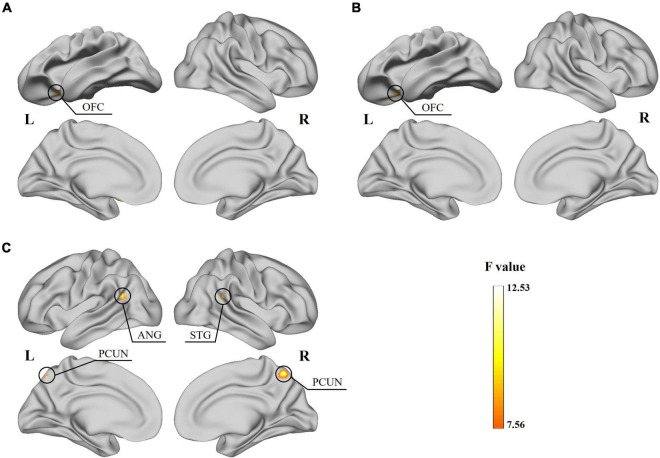
Results for the significant subtype and frequency interaction effect obtained from the two-way ANOVA. **(A)** Significant effect of the static fALFF on the left OFC surface. **(B)** Significant effect of the dynamic mean of fALFF on the left OFC surface. **(C)** Significant effect of the dynamic SD of ReHo on the right STG, bilateral PCUN and left ANG surface.

The two-way ANOVA showed a significant subtype × frequency interaction effect on the static fALFF in the left orbital-frontal gyrus (OFC) (Figure 2A and Table 2) and no significant difference in the static ReHo. In the analysis of dynamic indices, the ANOVA model showed interaction effect on dynamic mean of fALFF value in the left OFC (Figure 2B), dynamic SD of ReHo value in the right superior temporal gyrus (STG), bilateral precuneus (PCUN) and left angular gyrus (ANG) (Figure 2C).

**TABLE 2:** Significant results for the subtype and frequency interaction effect and the post-hoc analysis.

Functional Index	Region	L/R	Peak MNI coordinates			F	Cluster size (mm^2^)	Group difference^a^			
			**x**	**y**	**z**			**Slow-5**		**Slow-4**	
								**F**	**Sig.**	**F**	**Sig.**
Static fALFF	OFC	L	−24	18	−21	11.92	189	14.47	<0.001	0.37	0.691
Dynamic mean fALFF	OFC	L	−24	18	−24	12.14	189	15.40	<0.001	0.06	0.942
Dynamic SD ReHo	STG	R	54	−42	18	12.53	324	13.23	<0.001	1.12	0.333
	PCUN	L/R	0	−63	57	12.26	324	4.72	0.012	7.05	0.002
	ANG	L	−39	−54	21	11.74	270	12.27	<0.0001	2.72	0.073

SD, standard deviation; OFC, orbital-frontal gyrus; STG, superior temporal gyrus; PCUN, precuneus; ANG, angular gyrus.

^a^ANOVA results of significant interaction effect regions among ADHD-C, ADHD-I and TD groups.

### 3.3 ROI-based *Post-hoc* analysis

**FIGURE 3:**
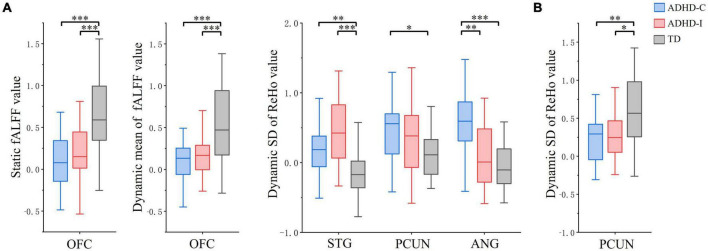
The comparation of ROIs among ADHD-C, ADHD-I and TD groups (Bonferroni corrected). **(A)** fMRI indices in the slow-5 band. **(B)** fMRI indices in the slow-4 band. Significant differences are marked by asterisks. *p < 0.05, **p < 0.01, ***p < 0.001.

**FIGURE 4:**
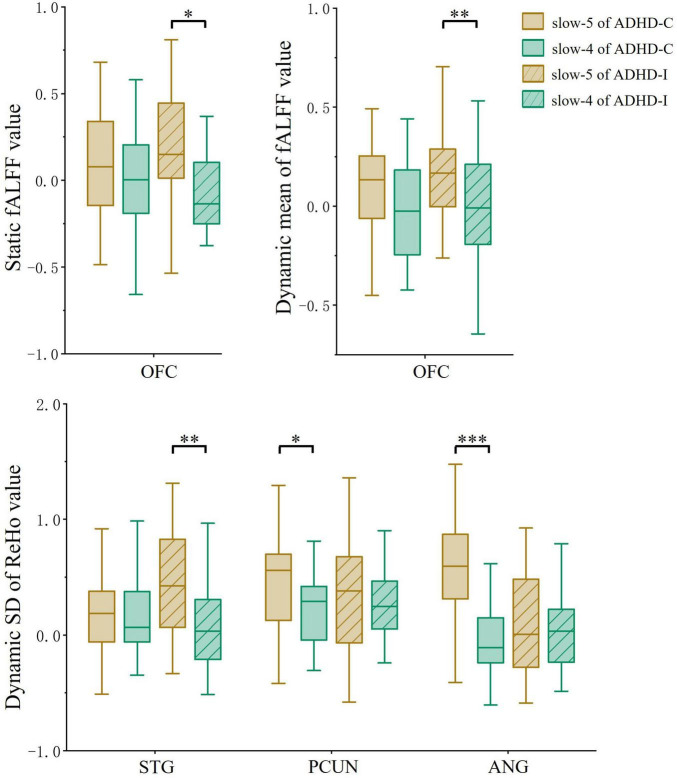
The comparation of ROIs between slow-5 and slow-4 bands. Significant differences are marked by asterisks. *p < 0.05, **p < 0.01, ***p < 0.001.

Regions showing subtype × frequency interaction effects were extracted as ROIs. A post-hoc analysis was performed on ROIs to investigate simple effects. Based on ROI analysis, rs-fMRI indices were compared among ADHD-C, ADHD-I and TD groups in the slow-5 band (Figure 3A) and show-4 band (Figure 3B). ADHD-C and ADHD-I both showed significantly decreased static and dynamic fALFF of OFC in the slow-5 band. Compared to TD, ADHD-C also showed increased dynamic ReHo SD values in the STG, PCUN and ANG, while ADHD-I only showed in the STG. Under the slow-4 band, both ADHD-C and ADHD-I exhibited decreased dynamic ReHo SD values in the PCUN. Furthermore, the ANG of ADHD-C and ADHD-I showed differences in slow-5. Additionally, ROI comparisons between slow-5 and slow-4 bands was shown in Figure 4. Compared with slow-4, ADHD-C showed increased indices of PCUN and ANG in slow-5, while ADHD-I showed increased indices of OFC and STG in slow-5.

### 3.4 Correlation and classification model

**FIGURE 5:**
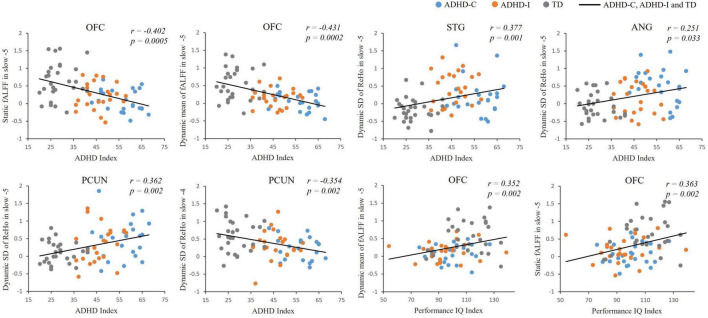
Partial correlation analysis between fMRI indices and clinical scores of ADHD-C, ADHD-I and TD.

**FIGURE 6:**
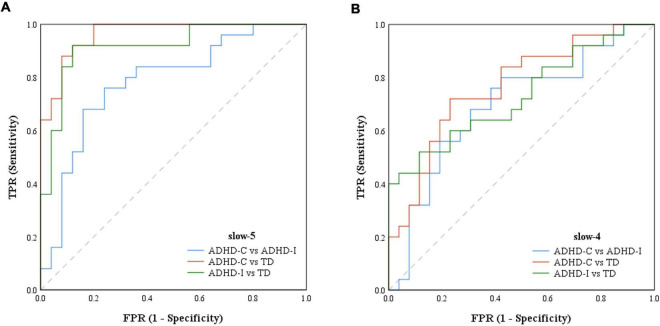
The ROCs of ADHD-C, ADHD-I and TD classifiers with features of ROIs. **(A)** ROC based on features in slow-5. **(B)** ROC based on features in slow-4.

The results of partial correlation analysis of three groups were shown in Figure 5. In the slow-5 band, all ROIs showed significant correlations with the ADHD index, while OFC also significantly correlated with the performance IQ index. In the slow-4 band, only dynamic SD ReHo of PCUN was negatively correlated with the ADHD index. The results showed that as static and dynamic mean indices decreased and the dynamic variabilities increased, the severity of disease increased. In addition, ROC curves were used to compare the classification efficiency of combined features from five ROIs in the slow-5 and slow-4 bands. The results indicated that features in slow-5 showed higher AUC and accuracy compared to features in slow-4 (Figure 6 and Table 3). Additionally, the features showed high classification efficiency in distinguishing between ADHD-C/ADHD-I and TD groups, while showed low performance in distinguishing between ADHD-C and ADHD-I groups.

**TABLE 3:** The results for classifying in ADHD-C, ADHD-I and TD groups with features of ROIs in slow-5 and slow-4.

Classifier	Features in slow-5			Features in slow-4		
	AUC	*p*-value	Accuracy(%)	AUC	*p*-value	Accuracy(%)
ADHD-C−ADHD-I	0.782	0.0006	76.47	0.695	0.0170	68.63
ADHD-C−TD	0.956	<0.0001	90.57	0.774	0.0006	77.36
ADHD-I−TD	0.907	<0.0001	88.89	0.729	0.0038	70.37

## 4 Discussion

To the best of our knowledge, researchers have not previously attempted to focus on comparing the neural activity patterns in specific divided subfrequency bands between different ADHD subtypes. We applied static and dynamic rs-fMRI measurements in subfrequency bands to examine the neural activity of ADHD-C, ADHD-I and TD. The results disclosed that significant subtype and frequency interaction effects were shown in the OFC, STG, PCUN and ANG. The post-hoc analysis showed that the intrinsic brain activity differences among three groups were mainly exhibited in the slow-5 frequency band and the interaction regions were significantly correlated with multiple clinical scores. Additionally, the features of significant interaction regions showed an advantageous diagnostic performance in slow-5 compared with those in slow-4.

Compared to healthy individuals, both ADHD-C and ADHD-I exhibited significantly decreased fALFF in the static and dynamic mean measurements of the OFC. Based on early investigations of the temporal and spatial structure of brain functional connections, that the state of brain activity varies over time (Hutchison et al., 2013; Allen et al., 2014; Yang et al., 2014). In recent years, an increasing number of studies have recognized that the intrinsic brain activity must be considered from the perspective of temporal features. The fALFF is a voxel-wised fMRI measure that provides information about the regional spontaneous amplitude intensity of brain activity within a network (Zou et al., 2008). The inferior frontal gyrus (orbital-frontal gyrus) has been reported to be involved in cognitive, motor control and sustained attention (Bush, 2011; Coffman et al., 2012). Furthermore, the orbital-frontal cortex is closely associated with executive functions, mediating top-down cognitive processes which operate in emotional and motivational contexts (Zelazo and Carlson, 2012; Salehinejad et al., 2021). This may reflect impaired activity of the motivational executive functions in both ADHD-C and ADHD-I. Research showed that ADHD individuals exhibit reduced activation in the left OFC during interference inhibition tasks (Cubillo et al., 2011). A previous study also showed that ADHD patients exhibit positive dynamic functional connectivity changes between the inferior frontal gyrus and regions of the cognitive control network following fMRI-neurofeedback training (Rubia et al., 2019). The abnormal activity of OFC may serve as the foundation for cognitive control deficits in individuals with ADHD, and the failure in cognitive control plays a crucial role in the main symptoms of ADHD.

In the slow-5 frequency band, ADHD-C showed increased dynamic SD of ReHo in the STG, PCUN, and ANG compared to TD, while ADHD-I only exhibited increased dynamic SD of ReHo in the temporal lobe. ReHo is a voxel-based measurement that assesses the similarity of the time series of a given voxel with those of its nearest neighboring voxels (Zang et al., 2004). We calculated SD of dynamic ReHo to measure the degree of dynamic variability in spontaneous neural activities over time. Cognitive function depends on dynamic interactions among large-scale neural systems (Bassett and Sporns, 2017). Most of the abnormal regions in our results overlapped with the default mode network (DMN) (Buckner et al., 2008). The DMN is active at rest but its activation decreases continuously during goal-directed behavior, which preserves the privilege of cognitive tasks by reducing unrelated stimulus activation (Raichle et al., 2001; Mantini et al., 2007). Some studies assumed that a decrease in the inhibition of the DMN is related to attention deficits (Castellanos and Proal, 2012). The increased SD of dynamic ReHo in the DMN reflected a high level of functional disruption, which may be associated with the disrupted balance within the neural network. In addition, ADHD-C exhibited higher dynamic SD of ReHo in the ANG compared to ADHD-C. The ANG acted as a multimodal integration center in reading, comprehension, spatial cognition and attention (Vannini et al., 2004) and showed reduced functional connectivity related to motor performance in ADHD (McLeod et al., 2014). A study examined the performance of ADHD patients in attention tasks revealed that the functional activity of ANG is related to cognitive performance (de Oliveira Rosa et al., 2020). Furthermore, research showed that the reduced functional connectivity between the PCUN and other components of the DMN is strongly associated with attentional deficits in ADHD (Castellanos et al., 2008). In slow-5, ADHD-C showed significant disturbances of functional activities in the PCUN and ANG, while ADHD-I did not. It may suggest that ADHD-C exhibits more severe symptoms of attention deficit than ADHD-I. A previous study also showed that ADHD-I performs better than ADHD-C in the test of attention network tasks (Oberlin et al., 2005). Additionally, compared to TD, both ADHD-C and ADHD-I showed decreased dynamic ReHo fluctuations in the PCUN in slow-4, which may reflect an excessive stability and ineffective activity. Our results suggested that ANG and PCUN, measured in slow-5, may be the key regions to distinguish ADHD-C from ADHD-I. The results might help explain the behavioral differences between ADHD-C and ADHD-I.

The subtype and frequency interaction effect analysis showed that abnormal fMRI indices of ADHD were not only related to disease factors, but also to specific frequencies. The subtype differences were mainly observed in slow-5 frequency band. Compared to the slow-4 band, functional signals in slow-5 tend to exhibit increased intensity or enhanced fluctuation. The results indicated that different frequency bands are associated with specific pathological states in ADHD-C and ADHD-I. This could suggest a high sensitivity of neural activity patterns in ADHD subtypes to the slow-5 frequency band, which contains more oscillation information in differentiating ADHD subtypes compared to slow-4. Furthermore, our results were consistent with a previous theory that brain regions showed different sensitivities to each frequency band: low-frequency oscillations (slow-5) have higher power, which is conducive to long-distance connections and large-scale neural network construction. Therefore, low-frequency oscillations more easily adjust the large default mode regions (Zuo et al., 2010; Han et al., 2011; Yu et al., 2014). High-frequency oscillations (slow-4) have lower power and are mostly related to the spatial structure of small neurons with short connections (Baria et al., 2011; Han et al., 2011). Moreover, it has been observed that the PCUN exhibits decreased ReHo in the slow-4 compared to the slow-5 in typically developing individuals. The functional connectivity showed a decreased tendency in the slow-4 band compared to the slow-5 band (Xue et al., 2014). These findings are similar to our results. The findings suggested that it is important to consider selecting sensitive frequency bands when detecting abnormal spontaneous brain activities in ADHD.

According to the correlative analysis among three groups, the spontaneous fluctuation indices of each group could reflect the serious trend in the development of the disease. For example, as the disease progressed, there is a gradual decrease in both static and dynamic mean fALFF of the OFC, and an increased variability in the dynamic ReHo of the PCUN, STG and ANG. Our results suggested that measuring neural fluctuation in specific frequencies may beneficial for understanding the neuropathological basis of ADHD and assist in future monitoring of disease progression. The results from dysfunctional regions applied to the classification showed that the diagnostic efficiency of features constructed in slow-5 was better than that in slow-4. The finding confirmed that the slow-5 frequency band contained more advantageous diagnostic information than slow-4 and it suggested that frequency factors should be considered when evaluating the intrinsic brain activities of ADHD. As more categorical features were derived from the dynamic indices, suggesting the temporal characteristics might be a powerful tool for the detection of pathological changes in different ADHD subtypes. Furthermore, the regions showing interaction effects could be potential neuroimaging markers representing the characteristics of ADHD subtypes.

Our study had some limitations that should be considered. First, our research did not include ADHD-HI patients because no ADHD-HI patients were included in this dataset. Second, the relatively small sample size may have limited the effect size of the study. As such, the replication efforts with larger sample sizes will be necessary to further confirm the robustness and reliability of the findings. Finally, this study focused on the assessment of ADHD in the resting state. More experiments of task state are needed to comprehensively evaluate the differences in brain functional activities of ADHD subtypes in different frequency bands.

## 5 Conclusion

Our study revealed that the intrinsic neural activities of ADHD subtypes were frequency-dependent and the differences were more evident in slow-5 than in slow-4 band. Furthermore, the abnormal indices were correlated to the trend of disease progression in each group. The study suggested that frequency factors should be considered when evaluating the neural substrates in ADHD subtypes. The frequency-dependent static and dynamic brain activities might provide potential neuroimaging biomarkers of ADHD subtypes and provide Supplementary Information for monitoring ADHD progressions.

## Data availability statement

The datasets presented in this study can be found in online repositories. The names of the repository/repositories and accession number(s) can be found below: the ADHD-200 Consortium.

## Ethics statement

The studies involving humans were approved by the Research Ethics Review Board of Institute of Mental Health, Peking University. The studies were conducted in accordance with the local legislation and institutional requirements. Written informed consent for participation in this study was provided by the participants’ legal guardians/next of kin.

## Author contributions

RC: Formal analysis, Investigation, Methodology, Software, Supervision, Validation, Writing−original draft, Writing−review and editing. YJ: Conceptualization, Methodology, Project administration, Supervision, Writing−review and editing. J-SZ: Formal analysis, Software, Supervision, Validation, Writing−review and editing. X-HW: Funding acquisition, Project administration, Resources, Supervision, Validation, Writing−review and editing. M-TZ: Resources, Software, Supervision, Validation, Writing−review and editing.
