# Supplementary material for: Frequency-specific static and dynamic neural activity indices in children with different attention deficit hyperactivity disorder subtypes: a resting-state fMRI study
Source: Front Hum Neurosci. 2024 Aug 9;18:1412572. doi: 10.3389/fnhum.2024.1412572 (PMC11345791; doi:10.3389/fnhum.2024.1412572)
Supplement: Supplementary file 1 [file Table_1.docx]

**Supplementary tables**

We varied the window length (32 TR/64 TR) to validate the reliability of the results. The results of dynamic indices with 32 TR window length have been included in Table S1 and Figure S1-S3. The results of dynamic indices with 64 TR window length have been included in Table S2 and Figure S4-S6.

**Table S1** | Significant results in dynamic indices for the subtype and frequency interaction effect and the post-hoc analysis (32 TR window length).

| **Functional Index** | **Region** | **L/R** | **Peak MNI coordinates** | | | **F** | **Cluster size (mm^2^)** | **Group difference^a^** | | | |
| --- | --- | --- | --- | --- | --- | --- | --- | --- | --- | --- | --- |
|  |  |  | **x** | **y** | **z** |  |  | **slow-5** | | **slow-4** | |
|  | | | | | | | | F | Sig. | F | Sig. |
| Dynamic mean fALFF | OFC | L | -24 | 18 | -24 | 12.24 | 189 | 12.59 | <0.001 | 0.59 | 0.557 |
| Dynamic SD ReHo | STG | R | 51 | -42 | 21 | 16.40 | 324 | 11.94 | <0.001 | 0.52 | 0.598 |
|  | PCUN | L/R | 0 | -63 | 57 | 11.76 | 270 | 4.54 | 0.014 | 5.38 | 0.007 |
|  | ANG | L | -39 | -57 | 21 | 11.32 | 216 | 10.78 | <0.001 | 1.69 | 0.191 |

*SD* standard deviation. *OFC* orbital-frontal gyrus, *STG* superior temporal gyrus, *PCUN* precuneus, *ANG* angular gyrus. ^a^: ANOVA results of significant interaction effect regions among ADHD-C, ADHD-I and TD groups.

**Table S2** | Significant results in dynamic indices for the subtype and frequency interaction effect and the post-hoc analysis (64 TR window length).

| **Functional Index** | **Region** | **L/R** | **Peak MNI coordinates** | | | **F** | **Cluster size (mm^2^)** | **Group difference^a^** | | | |
| --- | --- | --- | --- | --- | --- | --- | --- | --- | --- | --- | --- |
|  |  |  | **x** | **y** | **z** |  |  | **slow-5** | | **slow-4** | |
|  | | | | | | | | F | Sig. | F | Sig. |
| Dynamic mean fALFF | OFC | L | -24 | 18 | -24 | 10.40 | 189 | 14.77 | <0.001 | 0.06 | 0.940 |
| Dynamic SD ReHo | STG | R | 54 | -42 | 18 | 10.53 | 243 | 10.55 | <0.001 | 1.80 | 0.172 |
|  | PCUN | L/R | 0 | -60 | 57 | 10.73 | 324 | 5.00 | 0.009 | 6.70 | 0.002 |
|  | ANG | L | -42 | -51 | 21 | 11.28 | 189 | 12.50 | <0.001 | 2.76 | 0.070 |

*SD* standard deviation. *OFC* orbital-frontal gyrus, *STG* superior temporal gyrus, *PCUN* precuneus, *ANG* angular gyrus. ^a^: ANOVA results of significant interaction effect regions among ADHD-C, ADHD-I and TD groups.

**Supplementary figures**


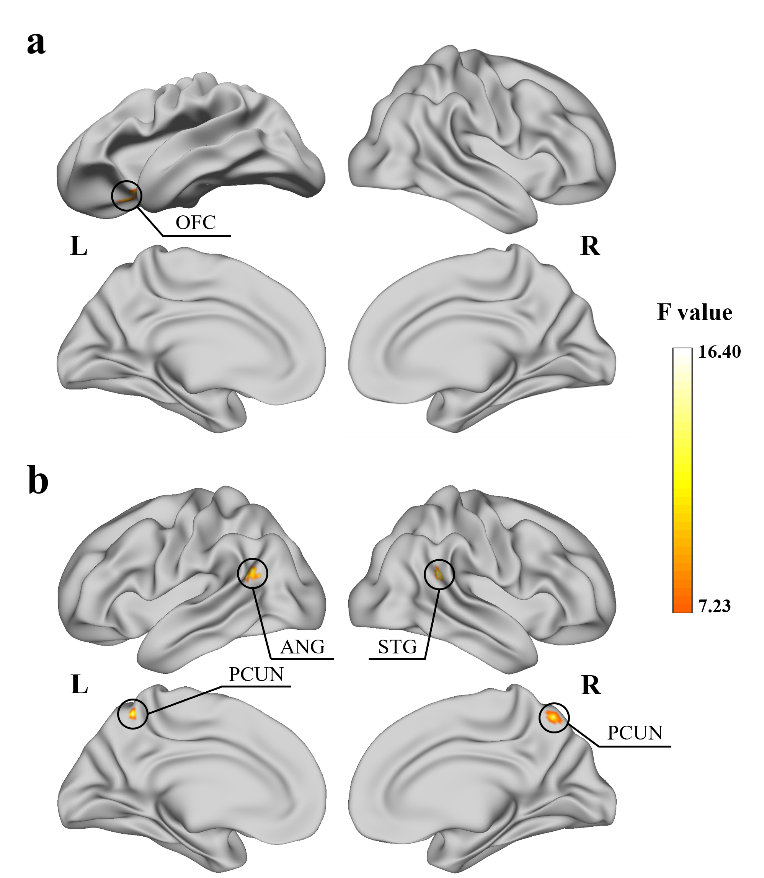


**Figure S1** | Results for the significant subtype and frequency interaction effect obtained from the two-way ANOVA (32 TR window length). (a) Significant effect of the dynamic mean of fALFF on the left OFC surface. (b) Significant effect of the dynamic SD of ReHo on the right STG, bilateral PCUN and left ANG surface.


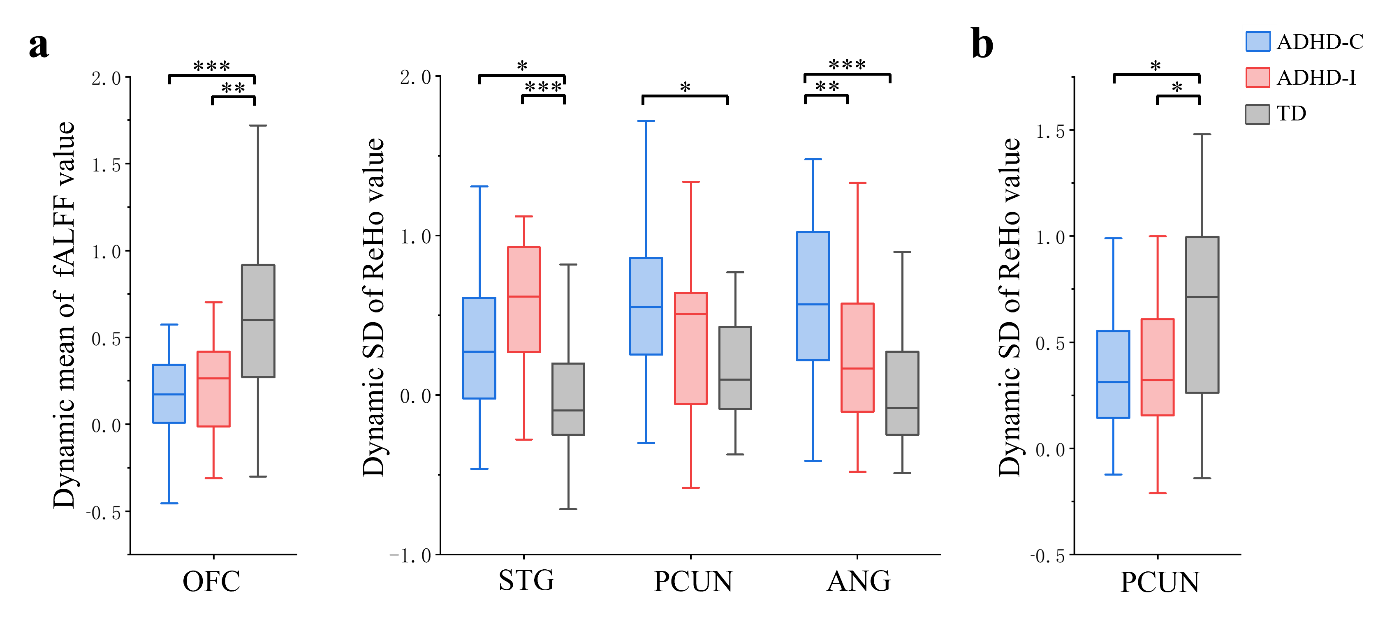


**Figure S2** | The comparation of ROIs among ADHD-C, ADHD-I and TD groups (Bonferroni corrected, 32 TR window length). (a) dynamic fMRI indices in the slow-5 band. (b) dynamic fMRI indices in the slow-4 band. Significant differences are marked by asterisks. *: p < 0.05, **: p < 0.01, ***: p < 0.001.


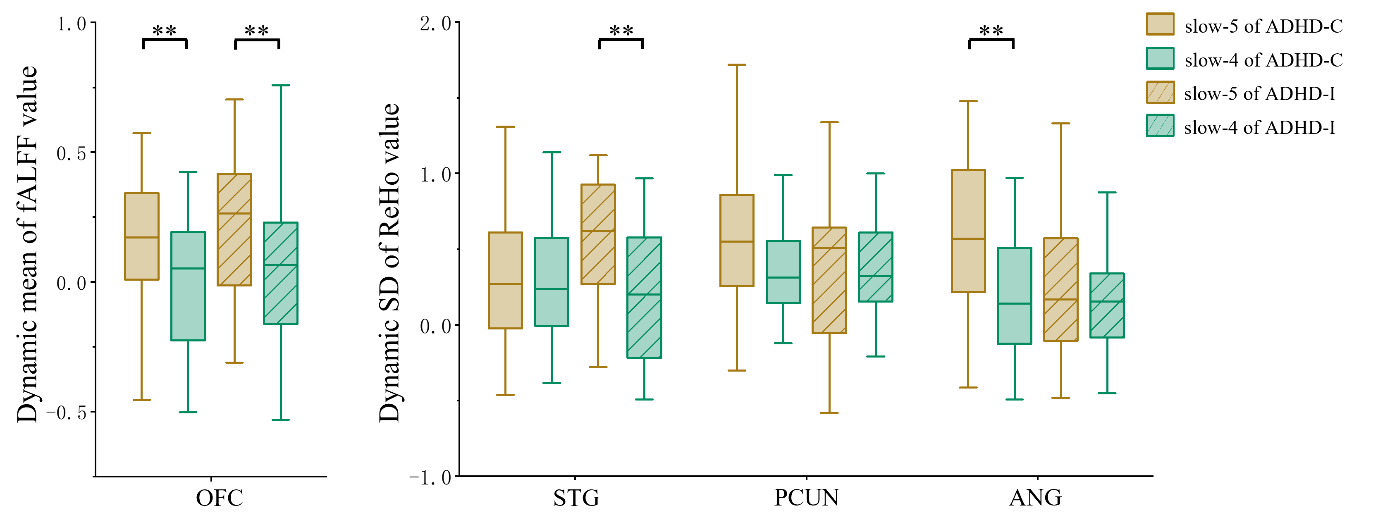


**Figure S3** | The comparation of ROIs between slow-5 and slow-4 bands (window length 32TR). Significant differences are marked by asterisks. *: p < 0.05, **: p < 0.01, ***: p < 0.001.


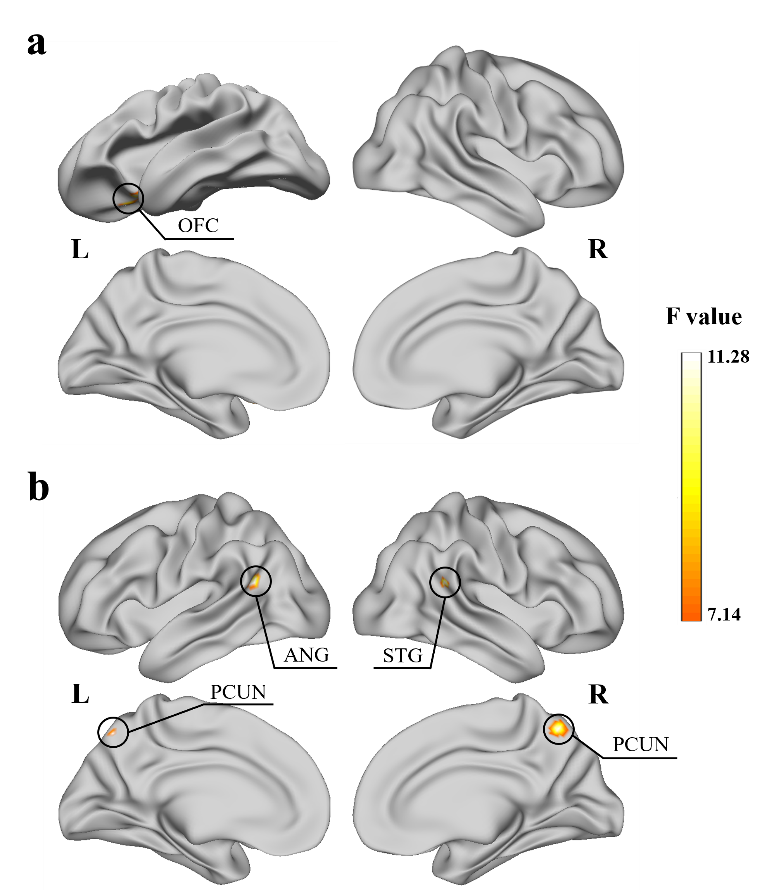


**Figure S4** | Results for the significant subtype and frequency interaction effect obtained from the two-way ANOVA (64 TR window length). (a) Significant effect of the dynamic mean of fALFF on the left OFC surface. (b) Significant effect of the dynamic SD of ReHo on the right STG, bilateral PCUN and left ANG surface.


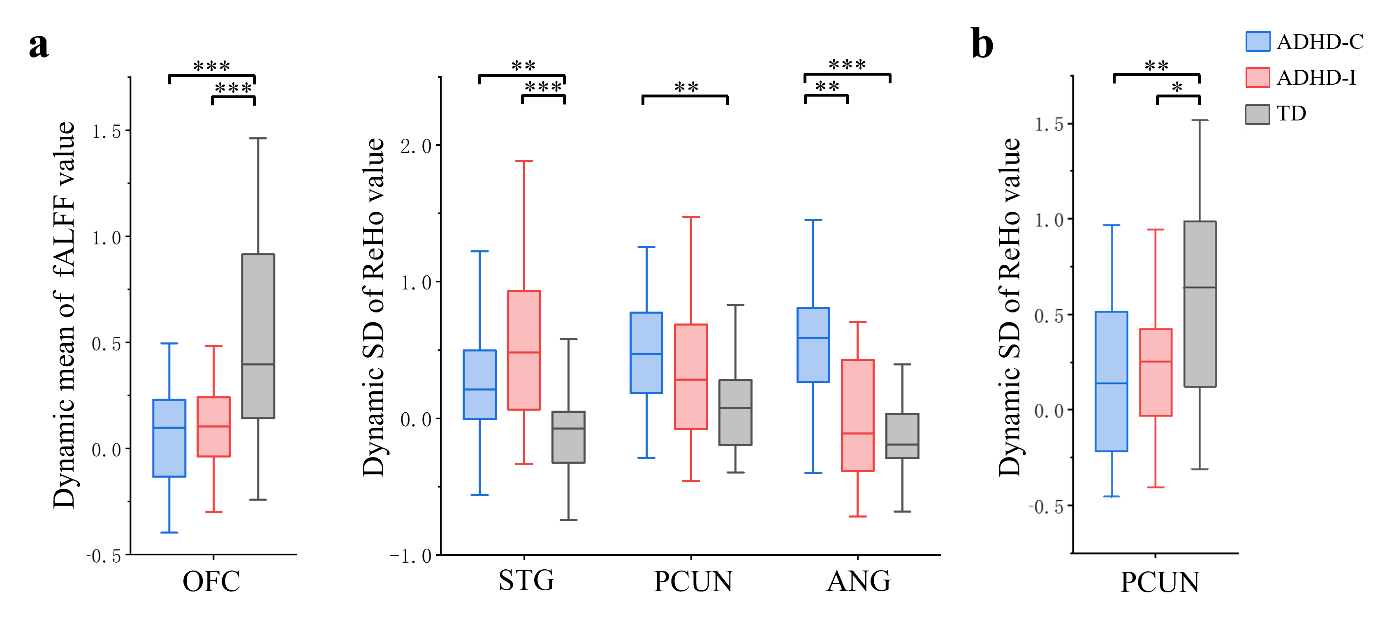


**Figure S5** | The comparation of ROIs among ADHD-C, ADHD-I and TD groups (Bonferroni corrected, 64 TR window length). (a) dynamic fMRI indices in the slow-5 band. (b) dynamic fMRI indices in the slow-4 band. Significant differences are marked by asterisks. *: p < 0.05, **: p < 0.01, ***: p < 0.001.


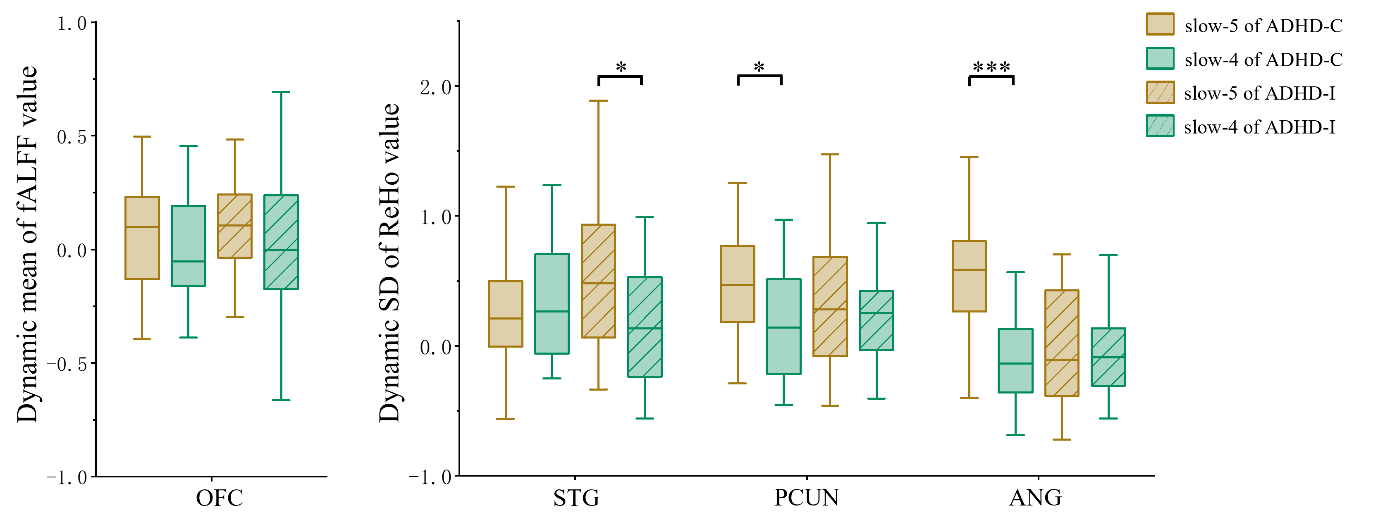


**Figure S6** | The comparation of ROIs between slow-5 and slow-4 bands (64 TR window length). Significant differences are marked by asterisks. *: p < 0.05, **: p < 0.01, ***: p < 0.001.
